# An Assessment of the Cariogenicity of Commonly Used Infant Milk Formulae Using Microbiological and Biochemical Methods

**DOI:** 10.1155/2011/320798

**Published:** 2011-11-14

**Authors:** Shweta Dixit Chaudhary, Mayur Chaudhary, Asha Singh, Sanket Kunte

**Affiliations:** ^1^Department of Pedodontics and Preventive Dentistry, SMBT Dental College, Amrutnagar, Ghulewadi, Sangamner, Maharashtra, Ahmednagar 422608, India; ^2^Department of Oral Pathology and Microbiology, SMBT Dental College, Amrutnagar, Ghulewadi, Sangamner, Maharashtra, Ahmednagar 422608, India; ^3^Department of Pedodontics and Preventive Dentistry, MGV Dental College, Maharashtra, Nasik 422003, India; ^4^Department of Pedodontics and Preventive Dentistry, Bharati Vidyapeeth Dental College, Maharashtra, Pune 411038, India

## Abstract

Dental caries is an important dental public health problem and is the most prevalent oral disease among children in the world. The present study was undertaken to evaluate and comparatively assess the change in plaque and salivary pH after ingestion of various commercially available infant milk formulae, and also to evaluate and comparatively assess plaque and salivary samples for change in colony-forming units of *Streptococcus mutans* caused due to their ingestion. 36 children in the age group of 1-2 years were fed with infant milk formulae three times a day for 21 days and results quantified. The present study revealed a highly significant increase in the levels of colony-forming units of *Streptococcus mutans* in both the plaque and salivary samples when assessed at baseline and after a period of 21 days, with the *t* value being 11.92 for the plaque samples and 11.66 for the salivary samples. It was also observed that all the test samples produced significantly lower plaque pH values than pre-feed pH. Based upon this study, further evaluation of the cariogenicity of infant milk formulae is recommended.

## 1. Introduction

Dental caries is an important dental public health problem and is the most prevalent oral disease among children in the world. The prevalence of dental caries has been of great interest for a long time and is a principal subject of significant research performed worldwide [1].

It is estimated that around 95% of the world's population is affected by dental caries [2].

Children suffer from many infectious diseases during the first three years of life around the time of eruption of the deciduous teeth. Early childhood caries, which is a combination of a child being infected with cariogenic bacteria and the frequent ingestion of sugar, is one of such diseases. Despite improvement over several decades, oral disease amongst children remains a serious problem [3].

Although dental caries has been declining globally in the general population, more so among older children, caries prevalence in younger ones has not shown a significant decline [3]. 

The caries prevalence in the primary dentition of preschool children in urban Pondicherry was found to be 44.4% with 26.7% in the maxillary arch and 36.9% in the mandibular arch [4]; the caries prevalence in preschool children in Hubli, Dharwad city was found to be 54.1%, [3] a very good indicator of the widespread hold of caries over our nation. 

Breast milk is a complete nutritious diet for neonates. However, some unfortunate children have to settle for infant formulas which contain lactose as the main ingredient [5].

Milk formulas can be categorized into three major groups comprising infant formula, follow-on formula and whole milk formula. The first group, infant formula, contains all the nutritional needs for infants during the first 4–6 months of life or until 12 months if used in conjunction with other infant foods. Infant formula can be classified by protein content into three subgroups as milk-based (cow's milk), soy-based, and protein hydrolysate formula. Soy-based formula is used for children with cow's milk allergy or lactose intolerance. Protein hydrolysate formula, in which the protein is hydrolysed into fragments of proteins and amino acids, is suitable for infants with protein sensitivity including galactosemia. Both soy-based and protein hydrolysate formula contain nonmilk extrinsic sugars such as sucrose and glucose syrup as carbohydrate resources. The second group, follow-on formula, is a modified cow's milk composition that covers the nutritional needs of infants during 6 months to 2 years of age. Follow-on formulas generally contain different amounts of casein. According to European Society for Pediatric Gastroenterology, Hepatology and Nutrition (ESPGAN), 1990, the manufacturers were allowed to add other sugars of no more than 20% of the total carbohydrate content in follow-on formula. The last group is whole milk formula, which is basically cow's milk with addition of necessary vitamins and mineral contents without any restriction of sugars added. It is recommended for children after the first year of age. Milk formulas have been implicated in the development of nursing caries, and controversy exists concerning the cariogenicity of milk [6]. 

Although widespread concern has been expressed about the content of baby bottles, particularly when they contain fruit juices or sugary solutions, information on the cariogenic potential of commonly used infant formulae is remarkably sparse [4]. 

With a goal to reduce the caries prevalence and to ultimately produce caries-free adolescents and adults, we need to stress upon the importance of primary prevention and increased restorative care [3]. 

Due to the high prevalence of dental caries, research must be carried out in the direction of identification of the children population at high risk of caries, so that a rigorous preventive program can be implemented [7]. 

High-risk group children with primary teeth decay should be identified and categorized, which in turn is useful to determine needs for restorations and to implement primary preventive procedures in the targeted group [3]. 

Hence it is our aim, through this study, to explore the cariogenic potential of the more commonly available infant milk formulae in relation to their ability to alter the plaque and salivary pH as well as to cause a change in the number of colony-forming units of *Streptococcus mutans *cultured from plaque and salivary samples. Thus, this study will help in creating awareness of the possible role of infant formulae in the development of early childhood caries in our country as well as to identify the groups at risk and institute timely primary preventive procedures. 

## 2. Aim and Objectives

1. To evaluate and comparatively assess the change in plaque and salivary pH after ingestion of various commercially available infant milk formulae.
2. To evaluate and comparatively assess plaque and salivary samples for change in colony-forming units of *Streptococcus mutans *caused due to ingestion of various commercially available infant milk formulae.

## 3. Materials and Methods

### 3.1. Sample Selection

The study included 36 children selected from three orphanages in Pune, India.

Informed consent was taken from the Managing Directors and the consulting pediatricians of the orphanages selected for the study. Approval of the Institutional Ethical Committee was obtained in accordance with the ethical guidelines prescribed by Central Ethics Committee on Human research (C.E.C.H.R.)

The subjects were chosen as per the following criteria.

**Inclusion Criteria**

1. Age between 12–24 months [8].
2. Caries free, dmf = 0.
3. Good general and dental health [9].

**Exclusion Criteria [9]**

1. Any systemic illness.
2. Children disliking infant milk formulae.
3. Antibiotic therapy in the past six months.
4. Drugs affecting salivary flow.
5. Children having xerostomia.
6. Lactose intolerance.

### 3.2. Materials Used for the Study

The following three commercially available infant milk formulae (IMF) were chosen for the study: Dexolac 2 follow-up infant formula (Wockhardt Ltd), Lactodex 2 follow-up infant formula (Raptakos Brett & Co Ltd), and Lactogen 3 follow-up infant formula (Nestlé India Ltd).

### 3.3. Presampling Measures

The selected formulae were prepared according to the manufacturers' instructions and utilized within half an hour of formulation. 5 level scoops of the formula were added to 150 mL of boiled water and mixed thoroughly to obtain a homogenous mixture.

The pH electrode of the pH meter was calibrated prior to the day of working for unprecedented accuracy across the measurement range. Two-point calibration was done using standard buffer solutions of pH 4 and pH 7. Calibration according to the temperature (37°C) was also done.

The selected children were randomly divided into three different groups consisting of 12 children per group.

The orphanage caretakers were asked to refrain from providing oral hygiene measures for the selected children for 24 hours prior to the sampling [8]. They were asked to avoid giving the children any foods except water for two hours prior to sampling. For this purpose, sampling was done early in the morning soon after the children got up from sleep. This provided us with at least 24-hour-old plaque [10]. 

### 3.4. Sampling Procedures

Prefeed plaque and salivary samples were collected on day one of the study.

### 3.5. Plaque Collection

Supragingival plaque (24-hour-old plaque using the plaque harvesting technique) was collected from buccal surfaces of alternating maxillary teeth using a spoon excavator (API German Stainless Steel Instruments) [11]. The tooth surfaces were patted with cotton to absorb saliva before collecting plaque to avoid salivary contamination. Collection was standardized as using four incisally/occlusally directed strokes on buccal surfaces of each tooth used for sampling. The collected pooled plaque was suspended in a sterile microcentrifuge tube with a snap-on lid, containing 50 microlitres of deionized water which was used as the dispersion medium. Deionized water was dispensed into the microcentrifuge tubes using an automatic micropipette [8]. 

### 3.6. Saliva Collection

Pooled unstimulated saliva was then collected using a Pasteur pipette [12] in a microcentrifuge tube with a snap on lid.

The prefeed readings for both plaque and salivary pH were taken using a standard pH meter along with a pH electrode as soon as the reading stabilized. Stuart's transport medium (STM) was then added to the prefeed samples which were stored in ice boxes for transportation. 0.95 mL of STM was used for the plaque samples. 0.90 mL of STM was used for the salivary samples. Hence, a dilution factor of 20 for plaque samples and 10 for salivary samples was obtained. 

### 3.7. Postfeed Sampling

Each child was fed with 150 mL of the formulated IMF within 10 minutes after taking the prefeed sample.

The children drank the formulated IMF using a medium sized stainless steel glass with a capacity of 300 mL. According to our observation, the children required an average of 4 minutes for their feed; that is, the teeth were in contact with the IMF for 4 min on an average.

As soon as they finished feeding, the postfeed samples were collected in a manner similar to that mentioned above for plaque and salivary sample collection. The postfeed samples were then used for pH monitoring at varying intervals as follows: immediate pH reading, 2 min, 5 min, 10 min, 15 min, 20 min, 25 min, 30 min, 40 min, 50 min, and 60 min.

Throughout the tests, a standard buffer solution of pH 7 was used, to ensure that the calibration of the pH meter remained constant.

Between each reading the electrode was cleaned with a stream of distilled water and placed in a standard buffer solution of pH 7.0. This ensured stable readings from the pH meter [13]. 

Thus the following pH measurements were done:

  Prefeed pH—defined as the pH recorded prior to feeding.
  Postfeed pH—defined as the pH recorded at varying intervals after feeding as follows: immediately after feeding, 2 min, 5 min, 10 min, 15 min, 20 min, 25 min, 30 min, 40 min, 50 min, and 60 min.
  Minimum pH—defined as the lowest pH recorded in the one hour period. It was recorded because the hydrogen ion production potential of food items has been related to the food's cariogenic potential.
  pH at the end of one hour—defined as the post feed plaque pH recorded at 60 min past the time of post feed plaque sampling.

And the following was computed:

  The maximum pH drop—defined as the difference between initial prefeed plaque pH and the minimum plaque pH obtained. It was calculated to account for the initial plaque pH of each subject.
  The pH drop at the end of one hour—defined as the difference between the prefeed plaque pH and the postfeed plaque pH recorded at 60 min past the time of initial plaque sampling. It was calculated to account for alterations in plaque acidogenicity caused by the sampling process [8].

After the one-hour monitoring, STM was added to the microcentrifuge tubes.

The prefeed samples were used for culturing. The inoculation (plating) of each sample was done within 6 hours of collection, on Mitis Sucrose Bacitracin Agar (Mitis Salivarius Agar Base + 2% bacitracin [i.e., 2 mg for every 100 gm] + 20% sucrose) [14] at 37°C for 72 hours in an anaerobic environment in a McIntosh Filde's jar, placed in an incubator. The colony-forming units of *Streptococcus mutans *were counted using the surface count method. Surface count method is generally used with opaque media in which a colony count is performed by spreading a known volume of suspension on the surface of a plate. The main advantage of this method is the ease of counting the colonies. 

The children were given servings of infant milk formulae thrice a day, keeping the remaining feeding schedules constant for three weeks [15]. On the 22nd day, prefeed and postfeed plaque and salivary samples were collected in a manner similar to that of sample collection on the first day. The prefeed plaque and salivary pH were noted and the postfeed plaque and salivary samples were monitored over an hour at the same intervals mentioned above to have a second set of pH readings and thus minimize any errors in the pH readings. 

These samples were transported under STM to the Microbiology Laboratory, and the postfeed samples were cultured on MSB agar at 37°C in an incubator for 72 hours. The colony count was performed.

There were no drop outs for the duration of the study.

### 3.8. Colony Counting

The volume of sample deposited in any portion of the sections was known and facilitated calculation of the number of CFU/mL of sample dilution, and when the dilution factor was taken into account per mL of undiluted sample suspension, the counts were obtained for each.

  0.05 mL plaque sample + 0.95 mL STM, that is, dilution factor = 20.
  0.1 mL saliva sample + 0.90 mL STM, that is, dilution factor = 10.

Hence total colony count = number of colonies counted by the surface count method on the Petri dish × dilution factor × 100 (for 1 mL volume).

Hence for plaque sample = *x* × 20 × 100, and for saliva sample = *y* × 10 × 100.

## 4. Results

**Figure 1:**
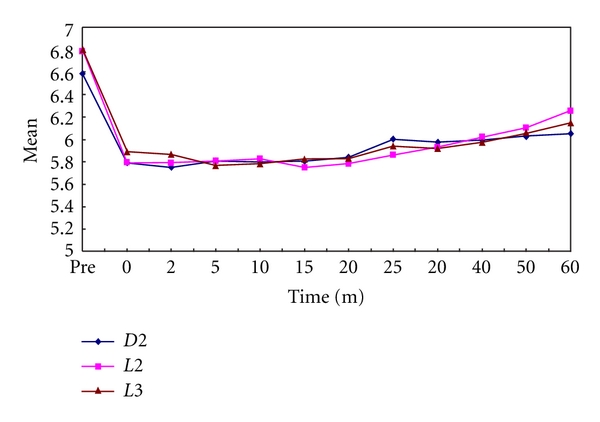
Variations in plaque pH values, before and after feed, between the three infant milk formulae on day 1 (D2: Dexolac 2, L2: Lactodex 2, L3: Lactogen 3).

**Figure 2:**
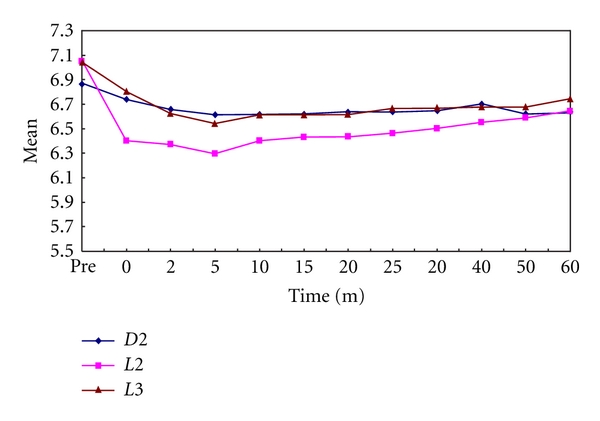
Variations in salivary pH values, before and after feed, between the three infant milk formulae on day 1 (D2: Dexolac 2, L2: Lactodex 2, L3: Lactogen 3).

**Figure 3:**
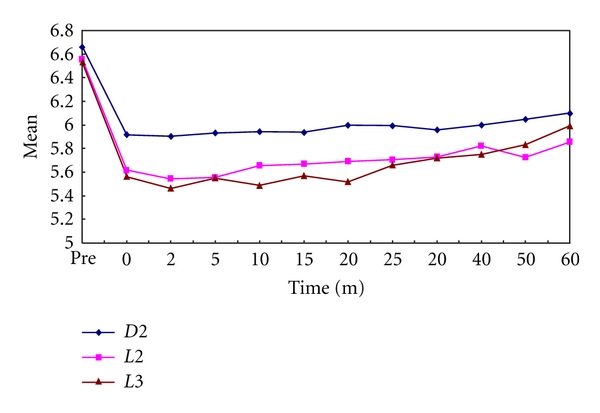
Variations in plaque pH values, before and after feed, between the three infant milk formulae on day 22 (D2: Dexolac 2, L2: Lactodex 2, L3: Lactogen 3).

**Figure 4:**
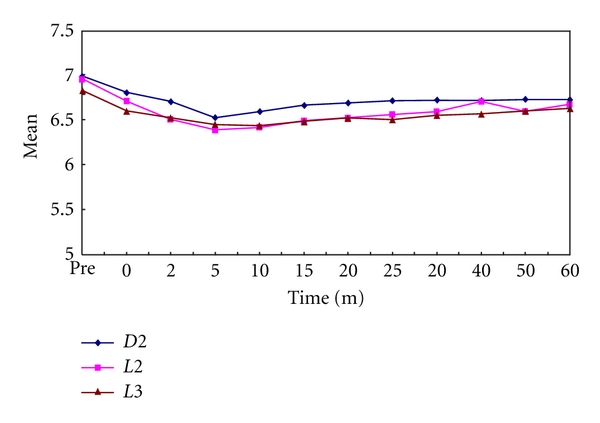
Changes in salivary pH values, before and after feed, between the three infant milk formulae on day 22 (D2: Dexolac 2, L2: Lactodex 2, L3: Lactogen 3).

In the present study, it was observed that all the test samples produced significantly lower plaque as well as salivary pH values than prefeed pH. The minimum pH reached in plaque samples after feeding on day 1 was 5.75, 5.85, and 5.69 for Dexolac 2, Lactodex 2, and Lactogen 3, respectively (Table 1, Figure 1). The minimum pH reached in salivary samples after feeding on day 1 was 6.62, 6.39, and 6.44 for Dexolac 2, Lactodex 2, and Lactogen 3, respectively (Table 2, Figure 2). After an adaptation period of 21 days, the minimum pH reached in plaque samples after feeding on day 22 was 5.9, 5.54, and 5.46 for Dexolac 2, Lactodex 2, and Lactogen 3, respectively (Table 3, Figure 3). After an adaptation period of 21 days, the minimum pH reached in salivary samples after feeding on day 22 was 6.56, 6.42, and 6.47 for Dexolac 2, Lactodex 2, and Lactogen 3, respectively (Table 4, Figure 4). Even though the drop was not as great as that for plaque, salivary pH levels could also serve as an indicator for the drop in pH levels upon consumption of a test food. All these results were found to be highly significant with a *t* value of 9.84 for plaque samples on day 1, 6.59 for salivary samples on day 1, 9.40 for plaque samples on day 22 and 6.92 for salivary samples on day 22 (Table 5). There was, however, no statistically significant difference between the three IMF when difference between prefeed pH and the minimum pH in plaque and salivary samples of children fed with Dexolac 2, Lactodex 2, and Lactogen 3 was computed, which indicates that the three IMF do not differ significantly in their ability to depress the plaque and salivary pH.

The present study also revealed an increase in colony-forming units of *Streptococcus mutans* in plaque samples from day 1 to day 22 as follows: from 45,167 to 66,500 in children fed with Dexolac 2, from 50,727 to 65,273 in children fed with Lactodex 2, and from 48,615 to 69,231 in children fed with Lactogen 3. A similar increase in salivary samples from day 1 to day 22 was found to be as follows: from 21,167 to 31,667 in children fed with Dexolac 2, from 23,909 to 31,000 in children fed with Lactodex 2, and from 23,154 to 33,154 in children fed with Lactogen 3 (Table 6). These results were found to be highly significant in plaque samples with a *t* value of 11.92 as also in salivary samples with a *t* value of 11.66 (Table 7). There was no statistically significant difference between the three IMF when evaluated for difference between the CFU of *Streptococcus mutans *in plaque and salivary samples of children fed with Dexolac 2, Lactodex 2, and Lactogen 3, which indicates that the three IMF do not differ significantly in their ability to alter the levels of *Streptococcus mutans*.

## 5. Discussion

*Early childhood caries* (ECC) is the new term for an infectious disease that affects the teeth of infants and toddlers. Its prevalence rate in developing countries has been shown to be as high as 70%. The cost of treating ECC is very high, and unfortunately, the risk factors for ECC point to various parenting practices [16].

Until more precise diagnostic criteria become available for the early lesion, the presence of *Streptococcus mutans *in high numbers, that is, >1% of the CFU, on a surface may be the foremost indicator of the future health of that surface [17]. 

Because of the irreversible nature of an established carious lesion, a true caries test on human subjects would be unethical. While several methods have been employed to test the cariogenic nature of foods, we do not have an acceptable model to investigate the caries process in the unusual conditions of nursing bottle caries. Food items have not been tested in pooling, stagnating conditions as seen in this disease. Therefore, there is a strong need to provide an experimental model that can better simulate the development of nursing caries and evaluate the potential role of infant milk formulas [10].

Despite interest in this subject for nearly 20 years, no universally accepted protocol has been developed to assess the cariogenicity of foods, nor has any one technique or group of techniques been adopted. Generally a combination of the following three methods has been accepted:

1. plaque pH profile,
2. ability to dissolve enamel,
3. production of caries in animals.

*Plaque sampling* is a technique which has provided useful information for various groups of investigators. Plaque is allowed to accumulate, with the subject abstaining from oral hygiene. After one or more days, the plaque is sampled from accessible sites on a large number of teeth by sequential scraping with an appropriate instrument. The pooled plaque sample is then dispersed in a diluent, and the pH is read with a microelectrode. 

Although there is a large body of literature on the different aspects of plaque acidogenesis, plaque pH alone has been questioned as a reliable measure of cariogenicity. Nevertheless, it has been claimed that measurements of pH have made it possible to examine dental plaque as a metabolic unit and to identify the Stephan pH response as an important indicator of caries activity [18]. 

In spite of the complexity of the fermentations which occur within plaque, the importance of looking at acid production by a sample food has been emphasized since the initial work by Stephan. Another point worth emphasizing is that saliva, by its effect on plaque pH, might have a significant influence on the microbial composition of plaque and that dietary components can significantly alter the microbial composition of human dental plaque [19]. 

One of the most consistent characteristics of people who experience little or no dental caries is the low acidity of their plaque. When examined in a standardized manner, plaque from caries resistant people (compared to that from caries susceptible subjects) exhibits a higher initial pH, a more modest fall in pH when challenged with sucrose, and a more rapid return to resting pH levels. Intra- or extraoral measurement of the pH of plaque samples exposed to sucrose provides a dramatic assessment of the risk for destruction of dental enamel. Specifically, the minimum of the Stephan curve is frequently considered as the criterion for the potential for caries initiation [20]. 

A food that does not lower the pH of plaque below a value of 5.7 is regarded as *“kind to the teeth”* and is given official recognition as such by the Swiss government [21].

The expression “safe for teeth” in German *“zahnshonend”* was defined by the Swiss Office of Health on 10th December, 1968 and on 22nd January, 1969 [22]. Experience and experimentation have shown that no product judged by the Swiss system as safe for teeth has been found to promote decay [10]. 

A statistically significant relationship between salivary levels of *Streptococcus mutans* and caries has been found by a number of researchers [7, 23–28]. A similar relationship between plaque levels of *Streptococcus mutans *and caries has also been found [17, 27, 29–31]. A longer prospective study has also recently reported the most significant predictors of caries at 3–5 years to be *Streptococcus mutans* [32]. 

The present study also reveals a highly significant increase in the levels of the colony-forming units of *Streptococcus mutans *in both the plaque and salivary samples when assessed at baseline and after a period of 21 days, with the *t* value being 11.92 for the plaque samples and 11.66 for the salivary samples. There is, however, no statistically significant difference between the CFU of *Streptococcus mutans *in plaque and salivary samples of children fed with Dexolac 2, Lactodex 2, and Lactogen 3, which indicates that the three IMF do not differ significantly in their ability to alter the levels of *Streptococcus mutans*. 

The present study also revealed that after an adaptation period of three weeks, it was found that there was a more pronounced fall in the plaque pH, alongwith a corresponding rise in the CFU of *Streptococcus mutans*. However, this was found to be statistically insignificant with a *t* value of 0.37 and a *P* value of 0.71. Birkhed et al., have earlier reported that the pH decreases were significantly more pronounced after the adaptation period than before, with 10% lactose and low fat bovine milk [33].

In the present study, it was observed that all the test samples produced significantly lower plaque pH values than prerinse pH. These results are in conjunction with the results observed by Sheikh and Erickson [10], Erickson et al. [34], Munshi et al. [35], Peres et al. [36], Al-Ahmari, and Joseph [37]. The salivary pH values were also significantly lower than the prerinse pH values. Even though the drop was not as great as that for plaque, salivary pH levels could also serve as an indicator for the drop in pH levels upon consumption of a test food. All these results were found to be highly significant with a *t* value of 9.84 for plaque samples on day 1, 6.59 for salivary samples on day 1, 9.40 for plaque samples on day 22 and 6.92 for salivary samples on day 22. There was, however, no statistically significant difference between the prefeed pH and the minimum pH in plaque and salivary samples of children fed with Dexolac 2, Lactodex 2, and Lactogen 3, which indicates that the three IMF do not differ significantly in their ability to depress the plaque and salivary pH.

As the initiation and progression of dental caries is a complex and multifactorial process, the evaluation of any one parameter can give no more than an indication of the potential cariogenicity of products. However, analytical studies evaluating the type of carbohydrate, inherent pH, and titratable acidity of these drink formulations, and in vivo studies on their ability to depress plaque pH, may give a fair insight into their potential to cause demineralization. It is generally accepted that foods leading to a lower plaque acidogenic response are safer for teeth than those which cause a more profound response. Therefore, the measurement of postchallenge plaque pH can, to some extent, be used as an indicator of the relative cariogenic potential of the drinks [13]. 

Foods can quickly be placed into two distinct categories: non- or hypoacidogenic foods and acidogenic foods. The former types of foodstuffs do not depress plaque pH in vivo to levels which have been proposed to bring about demineralization of tooth enamel. On the other hand, the latter foods cause acid to be produced by the plaque bacteria and might be projected to contribute to dental caries formation. Products showing little if any acidogenic potential allow them to be labeled “zahnschonend” or “friendly to teeth” [19]. 

For many years, investigators have sought to find an indicator (or set of indicators) for caries risk. The abundance of models that have been used for this purpose utilizes many different analytic approaches. However, one of the best indicators of caries development—independent of the study design—continues to be the presence of *Streptococcus mutans*. It is therefore likely that *Streptococcus mutans *levels in children might provide valuable information about the development, and possibly the severity of caries patterns [25]. 

If we indeed can identify the high risk susceptible individuals and offer them efficacious individual protection, something like a truncation of the risk distribution will occur when the elevated risk of the individuals is reduced to an acceptable level without affecting the risk of the remaining segment of the population. There are three basic prerequisites for a successful application of the high risk strategy in controlling dental caries. First, the occurrence of caries in the target population must be low enough to justify the effort and expense of identifying individuals who are believed to develop an unacceptably high number of cavities. Second, one must have accurate, acceptable and feasible measures for identifying the subjects with an unacceptably high risk. Third, the preventive efforts that aim at bringing down the elevated risk of these subjects should be based on measures that are effective and feasible [38]. 

The difficulty of predicting caries is not unexpected. The multifactorial etiology of dental caries makes it likely that even the most sophisticated risk models will be of limited value in predicting future caries development very accurately. It must also be remembered that even a perfect marker is only capable of predicting a person's future caries experience if the conditions on which the prediction is based remain stable [38].

Therefore, with overall dental caries rates in children decreasing and ECC prevalence remaining stable, ECC has become a proportionally larger contributor to dental caries in the pediatric population. To achieve a further decrease in dental caries, it is important to identify the individuals most at risk [39]. This would then facilitate the implementation of appropriate measures for the prevention of ECC and would prove to be a definite step, although in a long way, towards its eradication.

## 6. Recommendations

The present study has attempted to throw some light on the possible cariogenic potential of infant milk formulae. It underlines the need for the parent as well as the clinician to be fully aware of these effects of IMF and their possible role in ECC. A few recommendations are mentioned for the manufacturers of IMF as well as parents, so that in the near future, we may see a new generation of foods which, if not friendly to teeth in the strict sense, may be thought of as *“friendlier”* than their predecessors. 

### 6.1. Recommendations to Manufacturers of Infant Formulas

1. The composition of the infant milk formulae should be monitored on a regular basis.
2. The concentration of various carbohydrates like sucrose, lactose, and maltodextrins, which are responsible for the caries inducing potential of the infant milk formulae should be stated.
3. The cariogenic potential of the ingredients (carbohydrate and proteins) should be evaluated and labelled accordingly.

### 6.2. Recommendations to Parents by Pedodontists

1. Parents should be educated from time to time about the importance of diet in the development of caries (with regards to the type of carbohydrate).
2. Parents should be made aware of the fact that bovine milk is lower in sugar than formula milk and is higher in the protective factors, calcium and phosphorus [40]. Hence, they should be encouraged to provide bovine milk for their children when an alternative or an adjunct to breast milk is sought.
3. Parents should be discouraged from the frequent use of bottles for feeding, especially for prolonged periods at night.
4. Parents should be educated about the need for commencement of tooth brushing or cleaning the teeth with clean gauze or a clean muslin cloth, as soon as the first teeth erupt [41].

As a preventive measure, the dental professionals must thrive hard to make appropriate feeding of infants and children, a national movement supported by all health professionals. The parents and the pedodontist should work as partners in providing oral health care for children with ECC. 

## 7. Conclusion

It is envisioned that a lot of products may soon carry the label “Does Not Promote Tooth Decay”. Indeed, reasonable goals related to caries prevention would involve the development of means to either eliminate decreases in plaque pH when foods are eaten or accelerate the return towards less acidic levels [19].

Infant milk formulas and their process of manufacture are constantly changing in an attempt to come closer to human milk characteristics. Continued studies on the cariogenicity of infant milk formulae are important to assess the risks associated with their consumption [10]. The present study concludes that changes in plaque and salivary pH as well as colony-forming units of *Streptococcus mutans *after feeding with infant milk formulae when assessed as risk factors associated with caries implicate infant milk formulae in promoting caries in children. The conclusions of this study coupled with the understanding of a nursing child's behaviour should encourage the formulation and evaluation of less cariogenic infant milk formulae.

## Figures and Tables

**Table 1:** Computations of pH variations in plaque on day 1.

Infant milk formula	Prefeed pH	Minimum pH	pH at the end of one hour	Maximum pH drop	pH drop at the end of one hour	Average time taken to reach minimum pH (mins)
Dexolac 2	6.59 ± 0.59	5.75 ± 0.61	6.05 ± 0.61	0.84	0.54	2
Lactodex 2	6.83 ± 0.19	5.85 ± 0.56	6.29 ± 0.5	0.98	0.54	15
Lactogen 3	6.76 ± 0.2	5.69 ± 0.52	6.12 ± 0.46	1.07	0.64	5

**Table 2:** Computations of pH variations in saliva on day 1.

Infant milk formula	Prefeed pH	Minimum pH	pH at the end of one hour	Maximum pH drop	pH drop at the end of one hour	Average time taken to reach minimum pH (Mins)
Dexolac 2	6.87 ± 0.33	6.62 ± 0.46	6.64 ± 0.38	0.25	0.23	5
Lactodex 2	7.07 ± 0.28	6.39 ± 0.43	6.7 ± 0.29	0.68	0.37	5
Lactogen 3	7.04 ± 0.23	6.44 ± 0.51	6.69 ± 0.43	0.6	0.35	5

**Table 3:** Computations of pH variations in plaque on day 22.

Infant milk formula	Prefeed pH	Minimum pH	pH at the end of one hour	Maximum pH drop	pH drop at the end of one hour	Average time taken to reach minimum pH (Mins)
Dexolac 2	6.66 ± 0.39	5.9 ± 0.5	6.1 ± 0.4	0.76	0.56	2
Lactodex 2	6.56± 0.52	5.54 ± 0.71	5.86 ± 0.56	1.02	0.7	2
Lactogen 3	6.54± 0.61	5.46 ± 0.56	5.99 ± 0.58	1.08	0.55	2

**Table 4:** Computations of pH variations in saliva on day 22.

Infant milk formula	Prefeed pH	Minimum pH	pH at the end of one hour	Maximum pH drop	pH drop at the end of one hour	Average time taken to reach minimum pH (Mins)
Dexolac 2	7.03 ± 0.35	6.56 ± 0.42	6.77 ± 0.29	0.47	0.26	5
Lactodex 2	6.99 ± 0.39	6.42 ± 0.52	6.71 ± 0.43	0.57	0.28	5
Lactogen 3	6.87 ± 0.36	6.47 ± 0.44	6.67 ± 0.38	0.4	0.2	10

**Table 5:** Paired *t* test for the plaque and salivary samples between the prefeed pH and the minimum pH on day 1 and day 22 considering the three IMF together.

Sr. no.	Day of reading	For plaque samples		Significance	For salivary samples		Significance
		*t*	*P*		*t*	*P*	
1	Day 1	9.84	0.000	Highly significant	6.59	0.000	Highly significant
2	Day 22	9.40	0.000	Highly significant	6.92	0.000	Highly significant

**Table 6:** Variations in colony-forming units of *Streptococcus mutans*.

Sample	Plaque samples		Saliva samples	
	Day 1	Day 22	Day 1	Day 22
Dexolac 2	45,167 ± 8,156	66,500 ± 7,914	21,167 ± 4,019	31,667 ± 3,601
Lactodex 2	50,727 ± 9,931	65,273 ± 5,605	23,909 ± 4,549	31,000 ± 2,793
Lactogen 3	48,615 ± 6,449	69,231 ± 6,906	23,154 ± 3,105	33,154 ± 3,716

**Table 7:** Paired *t* test for the CFU of *Streptococcus mutans* in plaque and salivary samples on day 1 and day 22 considering the three IMF as a single group.

Sr. no.	For plaque samples		Significance	For salivary samples		Significance
	*t*	*P*		*t*		
1	11.92	0.000	Highly significant	11.66	0.000	Highly significant
